# Comparative analysis of treatment decision-making in patients with localized prostate and cervical cancer: what influences receiving surgery or radiotherapy?

**DOI:** 10.1007/s00520-024-08589-x

**Published:** 2024-05-29

**Authors:** Masanari Minamitani, Atsuto Katano, Tomoya Mukai, Shingo Ohira, Keiichi Nakagawa

**Affiliations:** 1 Department of Comprehensive Radiation Oncology, The University of Tokyo, 7-3-1 Hongo, Bunkyo-Ku, Tokyo, 113-0033 Japan; 2 Department of Radiology, The University of Tokyo Hospital, 7-3-1 Hongo, Bunkyo-Ku, Tokyo, 113-8655 Japan; 3 Department of Psychology, Fukuyama University, 985-1Higashimura-Machi, SanzoFukuyama-City, Hiroshima 729-0292 Japan

**Keywords:** Treatment decision-making, Psychosocial factors, Surgery vs. radiotherapy, Prostate cancer, Cervical cancer, Information sources in healthcare

## Abstract

**Purpose:**

This study focused on identifying the factors influencing the decision-making process in patients with localized prostate and cervical cancer in Japan and specifically examining the choice between surgery and radiotherapy.

**Methods:**

Patients with specific cancer stages registered with a healthcare research company for whom radical surgery or radiotherapy was equally effective and recommended participated in this cross-sectional online survey.

**Results:**

The responses of 206 and 231 patients with prostate and cervical cancer, respectively, revealed that both groups relied heavily on the physicians’ recommendations (prostate: odds ratio (OR) = 40.3, *p* < 0.001; cervical: OR = 5.59, *p* < 0.001) and their impression of radiotherapy (prostate: OR = 9.22, *p* < 0.001; cervical: OR = 2.31, *p* < 0.001). Factors such as hypertension (OR = 6.48, *p* < 0.05), diabetes mellitus (OR = 9.68, *p* < 0.05), employment status (OR = 0.08, *p* < 0.01), and impressions of surgery (OR = 0.14, *p* < 0.01) also played a significant role in patients with prostate cancer. In contrast, the specialty of the physician (OR = 4.55, *p* < 0.05) proposing the treatment influenced the decision-making process of patients with cervical cancer. Information sources varied between the two groups: patients with prostate cancer were more inclined towards printed materials, whereas patients with cervical cancer were more inclined towards interpersonal relationships.

**Conclusion:**

Although several limitations, such as the sample and recall bias, were noted, this study emphasizes the role of psychosocial factors in the decision-making process and the requirement for tailored information sources.

## Introduction

Cancer has been the leading cause of death in Japan since 1981, and the age-adjusted incidence rate has continued to increase [1, 2]. The prevalence of prostate cancer, the most prevalent type of cancer among Japanese males, has shown a steady increase [2]. The prevalence of cervical cancer among Japanese women declined until the 1990s; however, its incidence has increased since then [2]. This increase in prevalence and mortality is projected to continue owing to the spread of human papillomavirus infection among young women and limited participation in screening programs [2]. Several radical treatment options, including surgery and radiotherapy, are available for prostate and cervical cancer.

The National Comprehensive Cancer Network and Japanese clinical practice guidelines for prostate cancer recommend two radical methods, prostatectomy and radiotherapy, for the treatment of localized prostate cancer [3, 4]. Although the clinical outcomes are analogous, adverse events varies among low- and high-risk patients [4]. Radical hysterectomy or radiotherapy (alone or in combination with chemoradiotherapy) has been recommended by the Japanese guidelines for the treatment of International Federation of Gynecology and Obstetrics (FIGO) stages IB, IIA, and IIB cervical cancer [5]. In 2010, 40.2% of patients received androgen depletion therapy, 32.0% of patients underwent radical prostatectomy, and 21.0% of patients received radiotherapy as the initial therapy for newly diagnosed prostate cancer in Japan [6]. Among the patients with FIGO stage IB2 cervical cancer, 79% underwent hysterectomy, whereas 19% received radiotherapy [5]. Among the patients with FIGO stage IIA2 cervical cancer, 59% underwent hysterectomy, whereas 39% received radiotherapy [5]. The rate of utilization of radiotherapy for both types of cancer was lower than that calculated using the Malthus program, which was developed to gauge the appropriate use of radiotherapy [7].

Effective decision-making plays a crucial role in the field of oncology [8]. Patients with cancer should ideally acquire ample information regarding the disease and comprehend the benefits, harms, and uncertainties associated with different treatment options before making a decision [8]. However, achieving this goal remains challenging [8]. The decision-making criteria used in oncology can be divided into three categories: decision maker-related characteristics, decision-specific criteria, and contextual factors [9]. Decision maker-related characteristics comprise the attributes of physicians and patients, decision-specific criteria comprise classical clinical factors, and contextual factors comprise crucial elements, such as the healthcare system, financial factors, culture, and religion [9]. Information needs among patients with cancer are high; however, these needs often remain unmet [10]. Treatment-related information is demanded most frequently [11], and shared decision-making (SDM) has been recommended in recent years; however, adequate decision-making is rarely achieved in Japan [12].

Therefore, this study aimed to identify the factors influencing decision-making in the field of cancer treatment by evaluating the psychosocial factors affecting treatment choices in patients with prostate and cervical cancer at stages for whom surgery and radiotherapy are recommended to an equal extent as local treatments.

## Methods

### Study design and participants

A cross-sectional online study was conducted by a research agency for data acquisition to collect information from prostate and cervical cancer survivors. Potential participants received an invitation via email to participate and completed an online questionnaire. The healthcare research company, named “Macromill Carenet,” in collaboration with affiliated agencies, has cataloged over 500,000 patients in their collective panels [13]. Approximately 5000 patients with prostate cancer and 6000 patients with cervical cancer were identified in the database [13].

The target sample size, determined by the available study budget, aimed to recruit approximately two hundred patients for each of the prostate and cervical cancer groups. These patients were selected based on specific cancer stages for which surgery and radiotherapy are considered equivalently effective and recommended according to the guidelines. Individuals aged 20–99 years who had received radiotherapy or undergone surgery as the initial radical treatment for T1-T4N0M0 prostate cancer, classified according to the 8th edition of the UICC TNM staging system, or FIGO IB, IIA, or IIB cervical cancer, classified according to the 2018 FIGO staging system, and consented to participate in the survey were included in this study [14, 15]. The study protocol was approved by the Institutional Review Board of the Graduate School of Medicine and Faculty of Medicine, The University of Tokyo (2019363NI).

### Questionnaire design

The questionnaire gathered information regarding the demographic characteristics of the participants, including sex, age, marital status, number of children, education level, household income, and employment status at the time of diagnosis. In addition, clinical characteristics, such as cancer stage, performance status, past medical history, and elapsed time (defined as the amount of time from cancer diagnosis to the completion of the questionnaire) were recorded. Furthermore, the specialty of the doctors who initially proposed the cancer treatment and the size of the hospital to which the first doctor was affiliated were also recorded. A five-point Likert scale (0, strongly disagree; 1, disagree; 2, neutral; 3, agree; and 4, strongly agree) was used to grade the psychological factors, such as their priorities, motivations, psychological and temporal capacities, self and family involvement in the decision-making process, family’s past treatment experiences, symptoms, financial burdens, impression of surgery and radiotherapy, information needs, and doctor-patient relationship, influencing the decision-making process (Table 2). The participants selected one of the following five options to grade the initial doctor’s recommended treatment and decision-making approach:

- The recommended treatment was a continuous variable (0, surgery was the optimal treatment option; 1, surgery was a better treatment option than radiotherapy; 2, neutral; 3, radiotherapy was a better treatment option than surgery; and 4: radiotherapy was the optimal treatment option).
- The decision-making approach was a categorical variable (0, the physician made all treatment decisions; 1, the physician explained only the treatment option that they considered optimal and obtained consent from the patient; 2, the physician explained multiple treatment options and obtained consent for the treatment option that they considered optimal; 3, the physician explained multiple treatment options and shared the treatment decision with the patient; and 4, the physician explained multiple treatment options and left the treatment decision to the patient).

The respondents were also instructed to rate the information sources and the extent of their utilization using a five-point Likert scale (0 = never, 1 = hardly, 2 = occasionally, 3 = often, and 4 = always).

### Statistical analysis

The prostate and cervical cancer groups were analyzed independently to elucidate the significant factors influencing the decision-making process. Both groups were divided into two subgroups, surgery and radiotherapy, based on the initial treatment pursued. Systemic therapies such as chemotherapy, hormone therapy, and immunotherapy were not considered. Categorical and continuous variables were evaluated using the χ2 test and t-test, respectively, to identify the differences in their characteristics across subgroups. Logistic regression analyses were conducted with the initial treatment as the dependent variable. All characteristics, except age at the time of responding to the survey, were included as independent variables using a forward stepwise regression method (likelihood ratio). Variables with Spearman’s correlation coefficient R > 0.8 were excluded from the regression analysis to avoid multicollinearity. The extent to which patients with prostate and cervical cancer relied on information sources during the decision-making process was evaluated using a t-test. All statistical analyses were performed using SPSS software (version 27). The significance level was set at 5%.

## Results

This survey was conducted between April 28 and May 13, 2020. The agency sent email invitations to 685 potential participants with prostate cancer enrolled in the prostate cancer panel and collected 206 valid responses. Email invitations were initially sent to 3,144 known patients with cervical cancer. Due to a low initial response rate, we expanded our recruitment efforts to ensure sufficient data collection. Additional email invitations were sent to 970,609 individuals across the entire panel to identify and include other cervical cancer patients who may not have been previously registered as such. This resulted in 231 valid responses that satisfied the eligibility.

Table 1 presents the characteristics of the survey participants. The average age of the patients with prostate cancer was 70.6 years, whereas that of the patients with cervical cancer was 47.0 years. In the prostate cancer group, 128 and 78 patients were categorized into the surgery and radiotherapy subgroups, respectively. No significant differences were observed between the subgroups except for medical history. In the cervical cancer group, 180 and 51 patients were categorized into the surgery and radiotherapy subgroups, respectively. The patients included in the radiotherapy subgroup tended to have been diagnosed more recently (*p* < 0.01) and have a higher educational level (*p* < 0.01).

**Table 1:** Participants’ characteristics at the survey

	Prostate cancer patients					Cervical cancer patients				
	Surgery (*N* = 128)		Radiotherapy (*N* = 78)		*p* value	Surgery (*N* = 180)		Radiotherapy (*N* = 51)		*p* value
Sex, n (%)										
Male	128	(100%)	78	(100%)		0	(0%)	0	(0%)	
Female	0	(0%)	0	(0%)		180	(100%)	51	(100%)	
Age at the survey, y					0.18					0.07
Mean (SD)	70.1	(6.8)	71.4	(7.5)		47.9	(11.7)	43.9	(13.9)	
Elapsed time, n (%)					0.78					< 0.01
Less than 5 years ago	73	(57%)	46	(59%)		63	(35%)	30	(59%)	
More than 5 years ago	55	(43%)	32	(41%)		117	(65%)	21	(41%)	
Marital status, n (%)					0.45					0.76
Single/ widowed/ divorced	14	(11%)	6	(8%)		82	(46%)	22	(43%)	
Married	114	(89%)	72	(92%)		98	(54%)	29	(57%)	
Number of children, n (%)					0.13					0.95
Zero	11	(9%)	12	(15%)		68	(38%)	19	(37%)	
Any	117	(91%)	66	(85%)		112	(62%)	32	(63%)	
Education, n (%)					0.22					< 0.01
High school or less	52	(41%)	25	(32%)		145	(81%)	31	(61%)	
College or more	76	(59%)	53	(68%)		35	(19%)	20	(39%)	
Past medical history, n (%)										
Hypertension	50	(39%)	42	(54%)	0.04	30	(17%)	9	(18%)	0.87
Hyperlipemia	32	(25%)	25	(32%)	0.27	26	(14%)	6	(12%)	0.63
Diabetes mellitus	20	(16%)	16	(21%)	0.37	15	(8%)	5	(10%)	0.74
Mental disorder	0	(0%)	3	(4%)	0.03	18	(10%)	7	(14%)	0.45
Other cancer	15	(12%)	10	(13%)	0.81	17	(9%)	2	(4%)	0.21

Abbreviation: *SD* Standard Deviation

Table 2 presents the results of the univariate analysis of factors potentially influencing the decision-making process. A significant difference was observed between the subgroups of the prostate cancer group in terms of the age at the time of diagnosis (surgery, 64.0 years; radiotherapy, 66.1 years; *p* = 0.03). No significant differences were observed between the subgroups of both groups in terms of cancer stages or performance status, household income level, and employment status. The specialty of the first physician who recommended the initial therapy (prostate, *p* < 0.05; cervical, *p* < 0.001) and the recommendations (prostate, *p*-value < 0.001; cervical, *p* < 0.001) were identified as significant psychological factors in both groups. Moreover, a favorable impression (prostate, *p* < 0.001; cervical, *p* < 0.001) and family history of radiotherapy (prostate, *p* < 0.05; cervical, *p* < 0.001) were also identified as significant factors in the radiotherapy subgroup. Furthermore, patients’ focus on maintaining the quality of life (Q1), integration of their will into the decision-making process (Q6), impression of surgery (Q12), information needs (Q14), and impression of the physician (Q17) differed significantly between the subgroups of the prostate cancer group. The motivation for pursuing treatment (Q3), integration of the families’ will into the decision-making process (Q7), family’s experience with surgery (Q8), and cancer-related symptoms (Q10) differed significantly among the patients with cervical cancer.

**Table 2:** Univariate analysis of potential factors influencing the decision-making process

	Prostate cancer patients					Cervical cancer patients				
	Surgery (*N* = 128)		Radiotherapy (*N* = 78)		*p* value	Surgery (*N* = 180)		Radiotherapy (*N* = 51)		*p* value
Age at the time of the diagnosis, y					0.03					0.96
Mean (SD)	64.0	(6.5)	66.1	(7.1)		38.6	(10.0)	38.7	(13.2)	
TNM Clinical stage (prostate), n (%)					0.39					
Clinical stage I(T1-T2a,N0,M0)	66	(52%)	43	(55%)						
Clinical stage II(T2b-T2c,N0,M0)	40	(31%)	27	(35%)						
Clinical stage III(T3-T4,N0,M0)	22	(17%)	8	(10%)						
FIGO Clinical stage (cervical), n (%)										0.91
Clinical stage IB(IB1/IB2)						94	(52%)	25	(49%)	
Clinical stage II(IIA1/IIA2)						45	(25%)	14	(27%)	
Clinical stage IIB						41	(23%)	12	(24%)	
Performance status, n (%)					0.09					0.51
0	110	(86%)	73	(94%)		135	(75%)	34	(67%)	
1	18	(14%)	5	(6%)		38	(21%)	15	(29%)	
2	0	(0%)	0	(0%)		5	(3%)	2	(4%)	
3	0	(0%)	0	(0%)		2	(1%)	0	(0%)	
Household income, n (%)					0.60					0.34
< JPY 4.000.000	20	(16%)	11	(14%)		60	(33%)	20	(39%)	
JPY 4,000,000-	66	(52%)	36	(46%)		66	(37%)	21	(41%)	
Unknown/ not answer	42	(33%)	31	(40%)		54	(30%)	10	(20%)	
Employment status, n (%)					0.24					0.49
Working	92	(72%)	50	(64%)		144	(80%)	43	(84%)	
Not working	36	(28%)	28	(36%)		36	(20%)	8	(16%)	
Specialty of the first physician who recommended the initial therapy, n (%)					0.01					< 0.001
Urologist	127	(99%)	71	(91%)						
Gynecologist						174	(97%)	40	(78%)	
Radiation oncologist	1	(1%)	7	(9%)		6	(3%)	11	(22%)	
Hospital scale, n (%)					0.62					0.25
Cancer center, university hospital	47	(37%)	26	(33%)		79	(44%)	27	(53%)	
Gereral hospital. Clinic	81	(63%)	52	(67%)		101	(56%)	24	(47%)	
Psychological factors										
(Q1)* I Focused on maintaining the quality of life during and after my treatment					0.01					0.91
Mean (SD)	3.2	(1.0)	3.5	(0.8)		3.0	(1.0)	3.0	(0.7)	
(Q2)* I Focused on continuing working during and after my treatment					0.90					0.53
Mean (SD)	2.7	(1.2)	2.7	(1.3)		2.7	(1.2)	2.8	(1.1)	
(Q3)* I was motivated to engage in my treatment					0.90					0.00
Mean (SD)	3.6	(0.6)	3.6	(0.7)		3.4	(0.8)	3.0	(0.9)	
(Q4)* I had enough psychological capacities to decide my treatment					0.14					0.73
Mean (SD)	3.1	(1.0)	3.3	(0.7)		2.5	(1.3)	2.4	(1.2)	
(Q5)* I had enough temporal capacities to decide my treatment					0.54					0.27
Mean (SD)	3.1	(0.9)	3.2	(0.9)		2.3	(1.3)	2.5	(1.1)	
(Q6)* My will was integrated into the decision-making process					0.03					0.58
Mean (SD)	3.3	(0.8)	3.5	(0.7)		2.7	(1.1)	2.6	(1.1)	
(Q7)* My family’s will was integrated into the decision-making process					0.30					0.01
Mean (SD)	2.0	(1.1)	1.8	(1.2)		1.6	(1.3)	2.2	(1.3)	
(Q8)* My family experienced a surgery in the past					0.06					0.01
Mean (SD)	1.4	(1.4)	1.8	(1.5)		1.4	(1.5)	2.1	(1.5)	
(Q9)* My family experienced a radiotherapy in the past					0.04					< 0.001
Mean (SD)	0.9	(1.2)	1.3	(1.4)		0.9	(1.3)	1.9	(1.6)	
(Q10)* I suffered of cancer-related symptoms					0.17					0.03
Mean (SD)	0.9	(1.0)	1.1	(1.1)		2.2	(1.4)	2.7	(1.1)	
(Q11)* I suffered of the financial burden					0.17					0.24
Mean (SD)	1.2	(0.9)	1.4	(1.1)		1.8	(1.4)	2.1	(1.4)	
(Q12)* I had a favorable impression on surgery					< 0.001					0.67
Mean (SD)	2.3	(0.8)	1.5	(1.1)		1.8	(1.1)	1.9	(1.2)	
(Q13)* I had a favorable impression on radiotherapy					< 0.001					< 0.001
Mean (SD)	1.5	(0.9)	2.6	(1.0)		1.2	(1.0)	2.0	(1.0)	
(Q14)* My information needs on cancer and treatment were high					0.00					0.71
Mean (SD)	3.1	(0.9)	3.5	(0.7)		3.0	(1.3)	2.9	(1.2)	
(Q15)* I had a good doctor-patient relationship					0.84					0.42
Mean (SD)	3.2	(0.8)	3.2	(0.7)		3.1	(0.9)	3.0	(0.7)	
(Q16)† Which treatment did you feel was recommended by the first physician?					< 0.001					< 0.001
Mean (SD)	0.7	(0.7)	2.7	(1.0)		0.4	(0.7)	2.3	(1.3)	
(Q17) Which was your decision-making approach? n (%)					0.02					0.21
The physician made all treatment decisions	5	(4%)	2	(3%)		39	(22%)	10	(20%)	
The physician explained only the treatment option that they considered optimal and obtained consent from the patient	55	(43%)	30	(38%)		111	(62%)	29	(57%)	
The physician explained multiple treatment options and obtained consent for the treatment option that they considered optimal	34	(27%)	13	(17%)		21	(12%)	10	(20%)	
The physician explained multiple treatment options and shared the treatment decision with the patient	19	(15%)	9	(12%)		7	(4%)	0	(0%)	
The physician explained multiple treatment options and left the treatment decision to the patient	15	(12%)	24	(31%)		2	(1%)	2	(4%)	

Abbreviations: *FIGO* International Federation of Gynecology and Obstetrics, *SD* Standard Deviation

Scale items of psychological factor questions: *(Q1)-(Q15): 0, strongly disagree; 1, disagree; 2, neutral; 3, agree; 4, strongly agree

†(Q16): 0, surgery was the optimal treatment option; 1, surgery was a better treatment option than radiotherapy; 2, neutral; 3, radiotherapy was a better treatment option than surgery; and 4: radiotherapy was the optimal treatment option

**Fig. 1:**
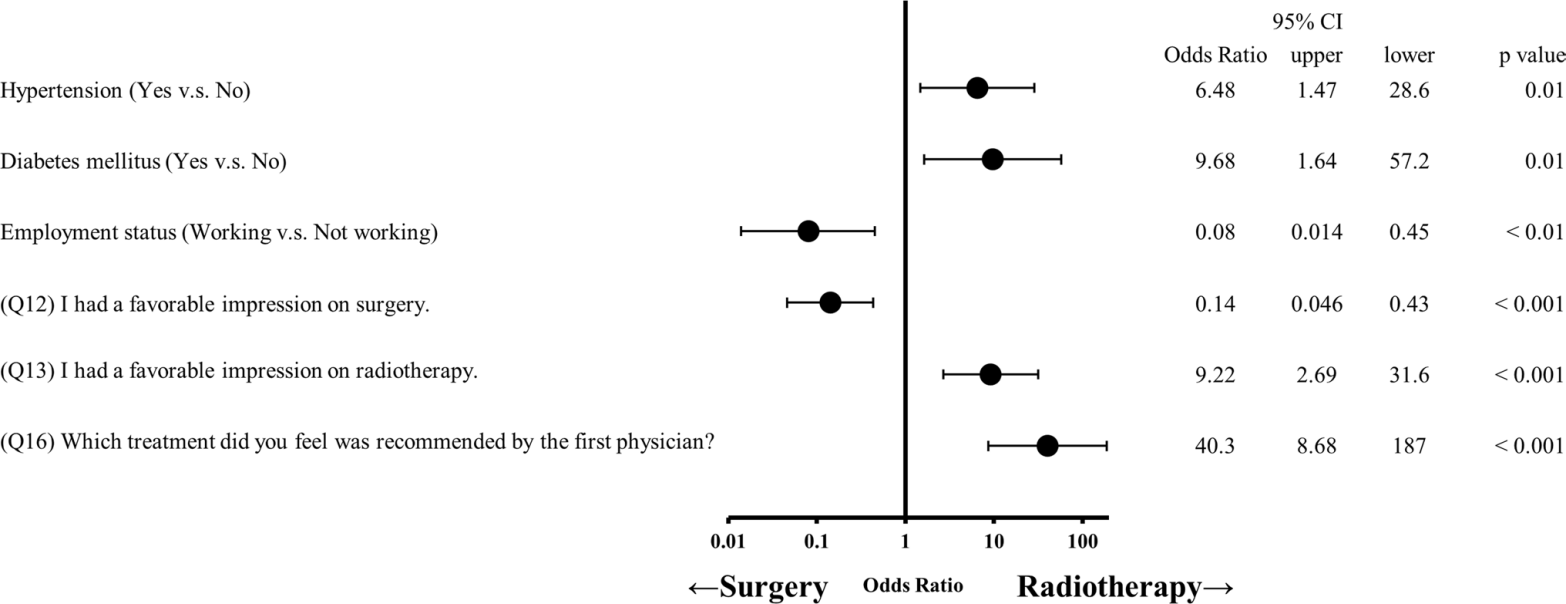
Odds ratio and 95% confidence intervals of the factors influencing treatment decision-making among patients with localized prostate cancer. CI, confidence intervals

**Fig. 2:**
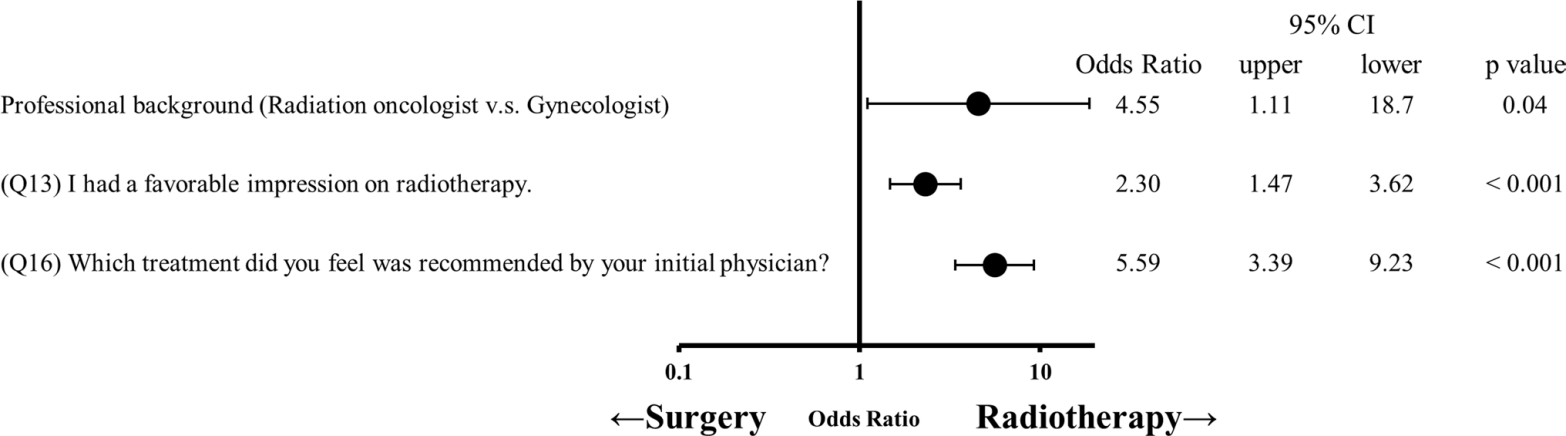
Odds ratio and 95% confidence intervals of the factors influencing treatment decision-making among patients with localized cervical cancer. CI, confidence intervals

Figures 1 and 2 show the results of the logistic regression analysis for each group. A positive impression of radiotherapy (prostate: odds ratio (OR) = 9.22, *p* < 0.001; cervical: OR = 2.31, *p* < 0.001) and physician’s recommendations for radiotherapy (prostate: OR = 40.3, *p* < 0.001; cervical: OR = 5.59, *p* < 0.001) showed significant association with the decision to pursue radiotherapy as the initial treatment in both groups. Hypertension (OR = 6.48, *p* < 0.05), diabetes mellitus (OR = 9.68, *p* < 0.05), unemployment (OR = 0.08, *p* < 0.01), and a negative impression of surgery (OR = 0.14, *p* < 0.01) were identified as significant factors for receiving radiotherapy in the prostate cancer group. A radiation oncologist proposing cancer treatment initially (OR = 4.55, *p* < 0.05) was significant in the cervical cancer group.

**Fig. 3:**
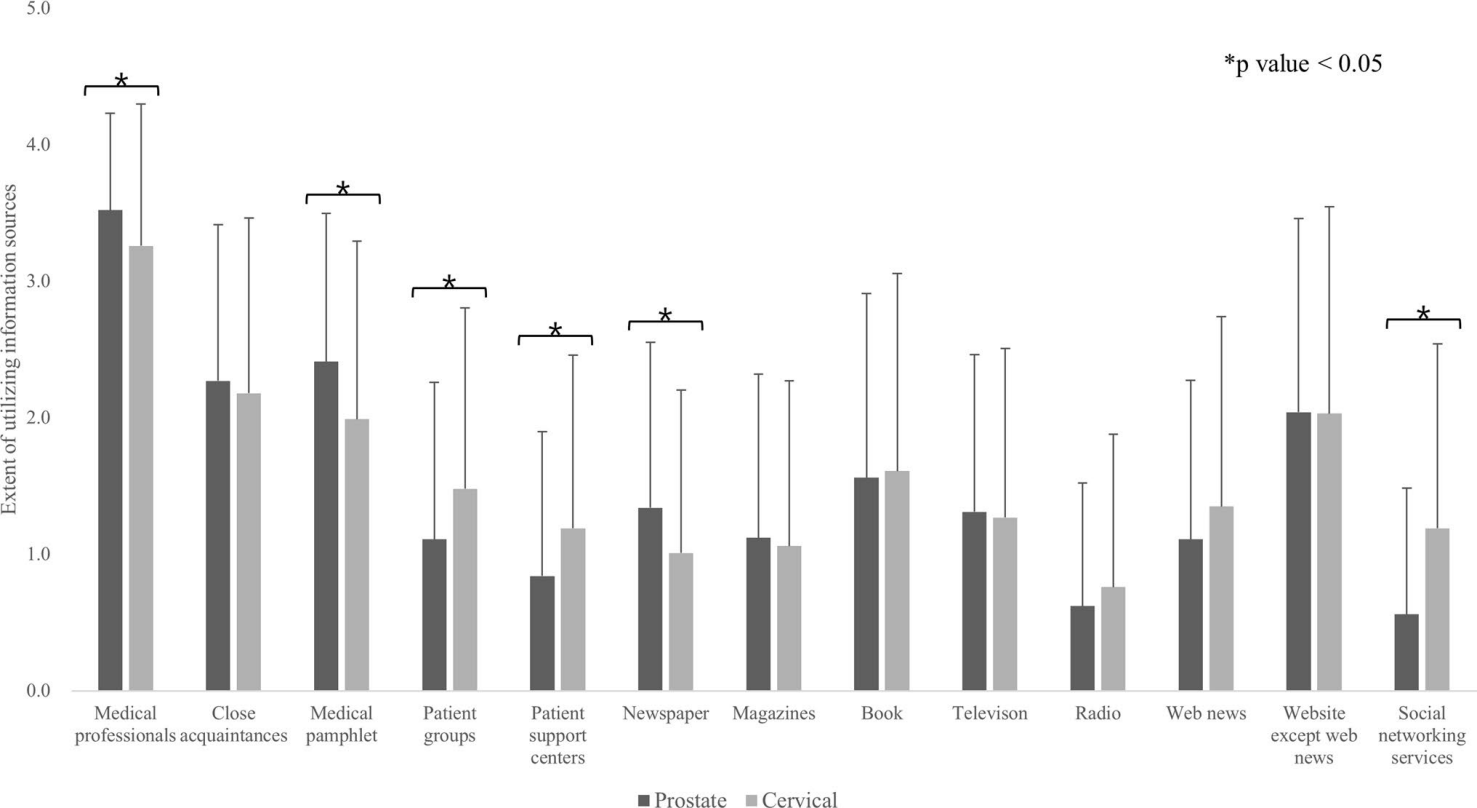
Comparison of the information sources for prostate and cervical cancer

Figure 3 presents the differences in information sources between the patients with prostate and cervical cancer. Patients often obtain information from medical staff, medical pamphlets, and close acquaintances, such as family and friends. Patients with prostate cancer favored obtaining information from medical professionals (3.5 vs. 3.3, *p* < 0.01), medical pamphlets (2.4 vs. 2.0, *p* < 0.01), and newspapers (1.3 vs. 1.0, *p* < 0.01). In contrast, patients with cervical cancer favored consulting organizations, such as patient groups (1.1 vs. 1.3, *p* < 0.01) and patient support centers (0.8 vs. 1.2, *p* < 0.01), as well as utilizing social networking (SNS) services (0.6 vs. 1.2, *p* < 0.001).

## Discussion

This study analyzed the factors influencing the decision-making process in patients with cancer. Patients with specific clinical stages of prostate and cervical cancer for whom radiotherapy and surgery are recommended as the initial radical treatments to an equal extent were included in this study. The recommendations of the physician-in-charge and a positive impression of radiotherapy significantly affected the decision-making process in both groups. The patients utilized information from healthcare professionals when making treatment decisions; however, the information sources varied between patients with prostate and cervical cancer.

Various factors are involved in the decision-making process of patients with cancer [9]. Trust in the physicians’ recommendations, concerns regarding side effects, the participation of the family in the decision-making process, and the ethnographic background influence the decision-making process in patients with prostate cancer [16, 17]. Physical comorbidities, such as hypertension and diabetes mellitus, were classified as decision-specific criteria in patients with prostate cancer in the present study. Impression of surgery and radiation therapy and physicians’ recommendations were classified as decision-maker-related criteria, and employment status was classified as a contextual factor, as indicated by a review published in 2020 [9]. Patients with prostate cancer who have multiple comorbidities often received radiotherapy in previous Japanese studies; thus, the association between physical complications and radiotherapy observed in this study is a reasonable result [18]. A study on employment among Japanese patients with cancer reported that treatment type (surgery, radiotherapy, or chemotherapy) had no effect on the rate of return to work [19]. The present study revealed no significant difference in the intention to return to work after treatment between the subgroups. Fewer employed patients selecting radiotherapy may be attributed to confounding factors that were not examined in this study, such as comorbidities and physical condition, rather than employment status. Psychological factors that were central to this study showed significant associations, particularly physicians’ recommendations, which showed the strongest relationship with the decision-making process (OR = 40.3) consistent with the findings of a previous study [17]. A previous report indicated that treatment recommendations for prostate cancer vary overwhelmingly among specialists, urologists, and radiological oncologists, which differs from the findings of the present study [20]. Notably, impression of surgery and radiotherapy were identified as significant factors influencing treatment choice in the present study.

A few studies have evaluated the factors influencing the treatment decision-making process among patients with cervical cancer. Some studies reported that financial barriers, knowledge of the cause of cervical cancer, and perceptions of the adequacy of specialists showed associations with the decision to undergo treatment, whereas age, comorbidities, cancer stage, and hospital type showed associations with delays in commencing treatment [21, 22]. The NCCN guidelines for patients with cervical cancer have listed hope, religious beliefs, feelings regarding certain treatments, and side effects as factors related to the decision-making process [23]. Physician’s specialty, impression of radiotherapy, and physicians’ recommendations, which are classified as decision-making criteria, were identified as significant factors in the present study [9]. The present study revealed a novel finding that the impression of radiotherapy influences the decision-making process similar to that in patients with prostate cancer. Several factors that were significant in the univariate analysis were excluded from the stepwise analysis. It was considered that these factors showed no significant associations with treatment choice due to confounding factors.

Almost all patients with cancer desire extensive and specific information regarding their illness [24]. However, approximately 40% of the information needs of the patients remain unmet [25]. Patients with prostate cancer patients had higher information needs than those with cervical cancer in the present study (3.2 ± 0.9 v.s. 3.0 ± 1.2, *p* = 0.01); however, the difference was small (Question: My information needs on cancer and its treatment were high). A large study revealed that unmet information needs were unaffected by sex or age; however, there was no concrete consensus [25]. The information most needed at the time of decision-making was treatment-related information (43.8%), followed by cancer-specific information (15.3%) [11]. Medical professionals were most frequently utilized as information sources, followed by close acquaintances, medical pamphlets, and web pages other than web news in the present study. A review conducted in the 2000s revealed that printed materials (35%), health professionals (27%), media (19%), interpersonal (12%), and organizational/scientific (8%) were useful, whereas a study from Singapore conducted in 2018 reported that doctors (52%), healthcare specialists (19%), and printed materials (11%) were prioritized [11, 26]. A Japanese survey that targeted individuals without cancer reported results that were similar to those of the present study, with medical professionals (56%), cancer support centers (44%), and close acquaintances (37%) being consulted more frequently [27]. The present study demonstrated that information sources differ significantly between patients with prostate and cervical cancer. Patients with prostate cancer were more inclined to receive information from printed materials, such as books and newspapers, in addition to medical staff, whereas cervical cancer patients tended to use SNS, in addition to interpersonal sources such as patient groups and cancer consultation support centers. SNS requires interpersonal communication. The preferred method of receiving information regarding prostate and gynecological cancer during post-treatment follow-up was as follows: in-person meetings, personalized reading, e-mail/internet, telephone calls, and personalized reading [28]. Information sources are highly susceptible to lifestyle; nevertheless, it was assumed that patients with cervical cancer are more inclined to interpersonal factors than patients with prostate cancer. Younger and female patients were more likely to rely on online sources for cancer information, thereby increasing the use of SNS among patients with cervical cancer [26]. Misinformation regarding health has become widespread in recent years, especially on social media; therefore, information should be organized based on the characteristics of each patient [29]. The findings of this study warrant further investigation.

## Limitations

This study has certain limitations. The first limitation of this study is sample bias, as this investigation focused on patients with cancer who had registered with a healthcare research company in Japan. The patient characteristics could differ from those of general patients with cancer. The second limitation was recall bias, as the responses of the study participants were based on their memory regarding the time of diagnosis and initial treatment. Approximately half of the patients were diagnosed > 5 years prior, and it may be difficult to recall as the patients are often overwhelmed by time-pressured decisions [30]. A cognitive bias wherein people often judge the past more positively than the present, that is, rosy retrospection, could have distorted this result [31]. This cognitive bias may be a critical limitation of this study. In addition, there was a noticeable interval between the completion of the survey and the publication of this paper. While it is acknowledged that public preferences and perceptions can change over a couple of years, the primary focus of this study was on the psychological impact at the time of treatment decision-making. Consequently, the publication delay is unlikely to substantially impact the validity of the study’s findings. The validity of the questions is the third limitation. This study aimed to compare the results of patients with prostate and cervical cancer; therefore, the same questionnaires were utilized, and questions regarding fertility were omitted, even for patients with cervical cancer. Radiotherapy targeting the pelvic region increases uterine dysfunction and leads to infertility [32]. The indications for radical trachelectomy, a treatment option that can preserve fertility, apply to patients with stage IA2 or IB1 cervical cancer with lesions ≤ 2 cm in diameter [5]. Our survey indicated that 12.6% (29/231) of the respondents, identified based on their stage (IB, due to the complexity of differentiating between sub-stages IB1 and IB2 in a patient-administered survey), age (under 36 years), and marital status (married), could potentially be candidates for such procedures. While we assessed the importance of quality of life during and after treatment, we did not specifically inquire about fertility, which may have influenced some patients’ treatment decisions. Although no differences in age, marital status, or number of children were observed, further research on women-specific features, focusing on QOL among the cervical subgroups, should be conducted. In addition to these limitations, unknown confounding factors may have influenced the results. Nevertheless, the findings of the present study indicate that psychosocial factors, including impressions of the treatment, play an essential role in decision-making in patients with cancer.

SDM is considered ideal for cancer treatment [33]. However, the approaches to introducing patient-centered care, such as SDM, are difficult to implement routinely in industrialized medicine [34]. Paternalism is particularly prevalent in Japan [35]. A recent survey of patients with prostate cancer and physicians revealed that almost half of the patients preferred SDM, whereas approximately 30% of physicians underestimated the willingness of the patients to be involved in treatment decisions [36]. The implementation rate of surgery and radiotherapy would change if the implementation of SDM is further enhanced in Japan and the patients’ intentions are reflected in treatment decisions. This will require further development of information sources and an increase in public awareness.

## Conclusion

This study examined the factors related to decision-making for the initial treatment, such as surgery or radiotherapy, of localized prostate and cervical cancer. The recommendations of the physician-in-charge and a positive impression of radiotherapy showed significant associations with the factors common to both types of cancer. Furthermore, hypertension, diabetes, employment status, and impression of surgery were significant factors for prostate cancer, whereas the specialty of the physician who first suggested the treatment was a significant factor for cervical cancer. Information sources vary for each type of cancer. As this study was a retrospective web-based survey with certain limitations, further research is warranted.

## Data Availability

No datasets were generated or analyzed during the current study.

## References

[1] Sung H, Ferlay J, Siegel RL (2021). Global cancer statistics 2020: GLOBOCAN estimates of incidence and mortality worldwide for 36 cancers in 185 countries. CA Cancer J Clin.

[2] Katanoda K, Hori M, Saito E (2021). Updated trends in cancer in Japan: incidence in 1985–2015 and mortality in 1958–2018—a sign of decrease in cancer incidence. J Epidemiol.

[3] NCCN Clinical Practice Guidelines in Oncology: Prostate Cancer (Version 2.2020) (2020) National Comprehensive Cancer Network. https://www.nccn.org/professionals/physician_gls/pdf/prostate.pdf. Accessed 28 Nov 2023

[4] The Japanese Urological Association (2016) Clinical practice guideline for prostate cancer. Version 2016. Medical Review Co., Ltd. Japan

[5] Ebina Y, Mikami M, Nagase S (2019). Japan Society of Gynecologic Oncology guidelines 2017 for the treatment of uterine cervical cancer. Int J Clin Oncol.

[6] Onozawa M, Hinotsu S, Tsukamoto T (2014). Recent trends in the initial therapy for newly diagnosed prostate cancer in Japan. Jpn J Clin Oncol.

[7] Jena R, Round C, Mee T (2012). The malthus programme — a new tool for estimating radiotherapy demand at a local level. Clin Oncol.

[8] Reyna VF, Nelson WL, Han PK, Pignone MP (2015). Decision making and cancer. Am Psychol.

[9] Glatzer M, Panje CM, Sirén C (2020). Decision making criteria in oncology. Oncol.

[10] Goerling U, Faller H, Hornemann B (2020). Information needs in cancer patients across the disease trajectory. A prospective study. Patient Educ Couns.

[11] Rutten LJF, Arora NK, Bakos AD (2005). Information needs and sources of information among cancer patients: a systematic review of research (1980–2003). Patient Educ Couns.

[12] Asai A, Okita T, Bito S (2022). Discussions on present Japanese psychocultural-social tendencies as obstacles to clinical shared decision-making in Japan. Asian Bioeth Rev.

[13] Macromill I Macromill, Inc. Website. https://www.macromill.com/. Accessed 28 Nov 2023

[14] Brierley JD, Gospodarowicz MK, Wittekind C (2017). TNM classification of malignant tumours.

[15] Lee SI, Atri M (2019). 2018 FIGO staging system for uterine cervical cancer: enter cross-sectional imaging. Radiology.

[16] Guan A, Shim JK, Allen L (2023). Factors that influence treatment decisions: a qualitative study of racially and ethnically diverse patients with low- and very-low risk prostate cancer. Cancer Med.

[17] Seaman AT, Taylor KL, Davis K (2019). Why men with a low-risk prostate cancer select and stay on active surveillance: a qualitative study. PLoS ONE.

[18] Yasui M, Sakaguchi M, Jikuya R et al (2020) Comparative effectiveness of surgery and radiotherapy for survival of patients with clinically localized prostate cancer: a population‑based coarsened exact matching retrospective cohort study. Oncol Lett 20. 10.3892/OL.2020.12013 10.3892/ol.2020.12013 PMC7475640 32934718

[19] Ito H, Hozawa A, Yamashita H (2015). Employment status among non-retired cancer survivors in Japan. Eur J Cancer Care (Engl).

[20] Fowler FJ, McNaughton Collins M, Albertsen PC (2000). Comparison of recommendations by urologists and radiation oncologists for treatment of clinically localized prostate cancer. JAMA.

[21] Tapera O, Dreyer G, Kadzatsa W (2019). Determinants of access and utilization of cervical cancer treatment and palliative care services in Harare, Zimbabwe. BMC Public Health.

[22] Shen S-C, Hung Y-C, Kung P-T (2016). Factors involved in the delay of treatment initiation for cervical cancer patients: a nationwide population-based study. Medicine (Baltimore).

[23] NCCN Guidelines for Patients: Cervical Cancer (2023) National Comprehensive Cancer Network. https://www.nccn.org/patients/guidelines/content/PDF/cervical-patient-guideline.pdf. Accessed 28 Nov 2023

[24] Jenkins V, Fallowfield L, Saul J (2001). Information needs of patients with cancer: results from a large study in UK cancer centres. Br J Cancer.

[25] Faller H, Koch U, Brähler E (2016). Satisfaction with information and unmet information needs in men and women with cancer. J Cancer Surviv.

[26] Chua GP, Tan HK, Gandhi M (2018). Information sources and online information seeking behaviours of cancer patients in Singapore. Ecancermedicalscience.

[27] Japanese Cabinet Office (2023) Public opinion survey on cancer control. https://survey.gov-online.go.jp/r05/r05-gantaisaku/. Accessed 28 Nov 2023

[28] Shea-Budgell MA, Kostaras X, Myhill KP, Hagen NA (2014). Information needs and sources of information for patients during cancer follow-up. Curr Oncol.

[29] Suarez-Lledo V, Alvarez-Galvez J (2021). Prevalence of health misinformation on social media: systematic review. J Med Internet Res.

[30] Edwards M, Davies M, Edwards A (2009). What are the external influences on information exchange and shared decision-making in healthcare consultations: a meta-synthesis of the literature. Patient Educ Couns.

[31] Mitchell TR, Thompson L, Peterson E, Cronk R (1997). Temporal adjustments in the evaluation of events: the “rosy view”. J Exp Soc Psychol.

[32] Wo JY, Viswanathan AN (2009). Impact of radiotherapy on fertility, pregnancy, and neonatal outcomes in female cancer patients. Int J Radiat Oncol Biol Phys.

[33] Beers E, Lee Nilsen M, Johnson JT (2017). The role of patients: shared decision-making. Otolaryngol Clin North Am.

[34] Montori VM (2022). Careful, kind care is our compass out of the pandemic fog. BMJ.

[35] Watanabe Y, Takahashi M, Kai I (2008). Japanese cancer patient participation in and satisfaction with treatment-related decision-making: a qualitative study. BMC Public Health.

[36] Schaede U, Mahlich J, Nakayama M et al (2018) Shared decision-making in patientswith prostate cancer in Japan: Patient preferences versus physician perceptions. J Glob Oncol 2018. 10.1200/JGO.2016.008045 10.1200/JGO.2016.008045 PMC6180796 30241183

